# Rapid Purification of Nuclei from Animal and Plant Tissues and Cultured Cells

**DOI:** 10.1100/tsw.2002.832

**Published:** 2002-06-07

**Authors:** John Graham

**Affiliations:** School of Biomolecular Sciences, Liverpool John Moores University, UK

**Keywords:** nuclei, tissue culture cells, liver, wheatgerm, OptiPrep^TM^, iodixanol, discontinuous gradient, isosomotic conditions

## Abstract

Nuclei are isolated by buoyant density banding in a discontinuous iodixanol gradient, under isoosmotic conditions. The low viscosity of the gradient allows the purification to be carried out at 10,000g in only 20 min. The method avoids possible damage to nucleoprotein complexes caused by hyperosmotic sucrose gradients. Although developed for mammalian liver the method can be applied (with or without minor modifications) to any tissue or cell type.

**METHOD TYPE:** extraction, isolation, purification and separation

**SUB METHOD TYPE:** centrifugation

## INTRODUCTION

Preparation of intact nuclei should be carried out in an environment that causes the least possible disruption to the nucleoprotein complexes. Ideally they should be isolated by isopycnic banding rather than by pelleting in order to avoid aggregation problems. Sucrose solutions however are not dense enough to band nuclei and although CsCl can provide solutions of sufficient density, its ionic strength causes disruption of nucleoprotein structure unless the chromatin is first fixed with formaldehyde.

Routine methods for purifying nuclei from mammalian sources use sedimentation of the organelles either from an homogenate or from a crude nuclear pellet through a 60% sucrose density barrier. Not only is the sedimentation of the particles very slow because of the high viscosity of the sucrose density barrier, the nuclei become severely dehydrated because of loss of water from their internal space due to the osmotic gradient across the membrane. This process may disrupt the macromolecular structures, which are normally highly hydrated.

Using iodixanol gradients nuclei can be isolated by isopycnic banding in an isoosmotic, nonionic environment[1,2]. Because the nuclei retain their normal hydration, their density is much lower than that in sucrose (1.20–1.22 against >1.32 g/ml). Buoyant density banding thus requires much lower concentrations of iodixanol; consequently the viscosity of the media is much lower, as are the centrifugation forces and times needed to purify the nuclei. The homogenate is adjusted to approx. $\rho$ = 1.14 g/ml and layered over two density barriers (A and B) of $\rho$ = 1.175 g/ml and $\rho$ = 1. 20 g/ml.

The protocol has been designed for mammalian liver; for other tissues or cultured cells see Note 1. For plant tissue sources see Note 2.

## MATERIALS AND EQUIPMENT

OptiPrep™ (60%, iodixanol, $\rho$ = 1.32 g/ml) – Axis-Shield PoC, AS, Oslo, Norway

Diluent: 150 mM KCl, 30 mM $MgCl_2$, 120 mM Tricine-NaOH, pH 7.8

Working Solution (WS) of 50% (w/v) iodixanol: mix 5 vol of OptiPrep™ 1 with 1 vol of Diluent

Homogenization Medium (HM): 0.25M Sucrose, 25 mM KCl, 5 mM $MgCl_2$, 20 mM Tricine-NaOH, pH 7.8

Centrifuge tubes for high-speed centrifuge (e.g., 40–50 ml polycarbonate)

High-speed centrifuge with swinging-bucket rotor (Sorvall HB4, Beckman JS13.1 or equivalent) to accommodate 40–50 ml tubes

Syringe with metal cannula (for underlayering)

## METHOD

1. Prepare two gradient solutions of 30 and 35% (w/v) iodixanol by diluting WS with HM (6 vol. + 4 vol. and 7 vol. + 3 vol, respectively).
2. Produce a homogenate of the tissue or cell using HM and either use this homogenate or a crude nuclear pellet (1000*g* for 10 min) resuspended in HM (see Note 3).
3. Mix equal volumes of the sample and WS and transfer 10–15 ml to a suitable centrifuge tube (40–50 ml) for a swinging-bucket rotor of a high-speed centrifuge (see Note 4).
4. Underlayer the sample with 10 ml of the 30% iodixanol and 5–10 ml of the 35% iodixanol and centrifuge at 10,000$g_{av}$ for 20 min (see Notes 5–7).
5. The band of nuclei at the 30–35% iodixanol interface (Fig. 1) of the total DNA. Phase contrast microscopy shows no discernible contaminants and 95% of the succinate dehydrogenase is recovered in the sample layer.

## NOTES

1. **Other tissues and cultured cells**. The density and/or the rate of sedimentation of nuclei from other tissues and from cultured mammalian cells may be different to those from mammalian liver. Thus it may be necessary to modulate either the centrifugation time or the density of the layers; nuclei from CHO cells for example band at a 25/30% interface[3]. The strategy has however found a wide application in the form described in this Protocol Article[4,5,6,7,8].

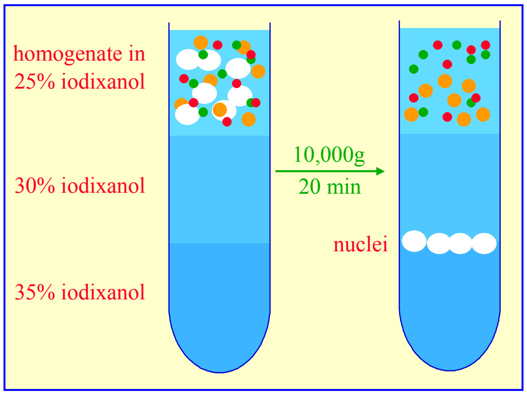

**FIGURE 1.** Purification of nuclei in a discontinuous iodixanol gradient.

2. **Plant tissues** may need some modification to the density and composition of the gradient to achieve optimal separation of nuclei. The osmolarity of the suspending medium (SM) is often higher: for wheatgerm 0.4 M sucrose, 25 mM KCl, 5 mM $MgCl_2$, 10 mM MES, pH 6.2 has been found to be optimal and the two layers should be prepared according to Table 1. The osmolarity of these solutions is 480–500 mOsm. 20 ml of a crude nuclear suspension is layered over 5 ml each of the gradient solutions. After centrifugation at 5,600*g* for 30 min the nuclei band at the lower interface, other organelles at the top interface and the starch granules pellet. Other variants of the technique include adding OptiPrep™ to the crude nuclear suspension to adjust the density of the suspending medium to $\rho$ = 1.234 g/ml and layering the $\rho$ = 1.167 g/ml layer on top. As with animal tissues and cells, the density of the gradient layers (and the centrifugation conditions) may require modulation to optimize the purification for specific types of plant tissue.

**TABLE 1**
**Gradient Solutions for Wheatgerm Nuclei**

| Density (g/ml) | OptiPrep™ (ml) | SM (ml) |
|---|---|---|
| 1.167 | 8.5 | 11.5 |
| 1.234 | 13.5 | 6.5 |

3. The principal means by which mammalian tissues and cells are homogenized are given in Refs. [9,10], respectively. Ideally the HM should have a composition as defined above. This may not be possible with some cultured cells. If the latter require the use of either a divalent cation-free solution or a hypoosmotic solution to effect efficient cell breakage, then adjust the composition of the homogenate to that of the HM once the cells have been homogenized.
4. The volume of the homogenate and density layers may be scaled up or down as required. If it is acceptable to allow the nuclei to pellet, then the densest layer may be eliminated. However banding them at an interface is a gentler option and avoids the aggregation problems associated with pelleting.
5. The rapid sedimentation rate of nuclei, compared to that of other particles present, ensures that only the nuclei are able to sediment through the 30% iodixanol layer at the time and rotor speed used. Other particles remain in the sample or at the sample/30% iodixanol interface (see Fig. 1).

6. With mammalian liver, the protocol can be carried out at g-forces as low as 5000*g* (for 20 min) without any significant reduction in recovery of nuclei.
7. Protease inhibitors (PMSF, leupeptin, antipain, aprotinin, etc.) may be included in any or all of the media at the operator's discretion.

## ACKNOWLEDGEMENTS

The author and TheScientificWorld wish to thank Axis-Shield PoC, AS, Oslo, Norway for their kind permission to adapt OptiPrep™ Application Sheet S8 in the preparation of this Protocol Article.
